# A Rare Case of Ipsilateral Scrotal Recurrence of Testicular Cancer After Radical Orchidectomy

**DOI:** 10.7759/cureus.51219

**Published:** 2023-12-28

**Authors:** Anna Akpala, Saloni Bhattacharyya, Adebiyi Damola, Richard Viney

**Affiliations:** 1 Urology, Queen Elizabeth Hospital Birmingham, Birmingham, GBR; 2 Urology, University of Birmingham, Birmingham, GBR; 3 Urology, George Elliot Hospital, Nuneaton, GBR

**Keywords:** recurrence tumor, high inguinal orchidectomy, hemi scrotum, testicular germ cell tumors, testicular mass

## Abstract

A lump in the testicle, painful or painless, could represent testicular cancer. Testicular cancer can be subdivided into germ-cell testicular cancer and sex cord-stromal tumors. A majority of testicular neoplasms are germ cell tumors (GCTs). GCTs are broadly divided into seminomatous and non-seminomatous germ cell tumors (NSGCTs) due to differences in natural history and treatment. Removal of the testis, also known as a radical orchidectomy, is often offered as part of the treatment for testicular cancer, which may be followed by additional medical treatment. It is not very common to have a recurrence of testicular cancer in the scrotum after a radical orchidectomy, and it is even rare to find this scrotal recurrence on the same side.

An extensive literature review showed only one recorded case of scrotal recurrence of NSGCTs after orchidectomy but on the contralateral side. Here, we report the first case of scrotal recurrence of NSGCT after radical inguinal orchidectomy on the same side in a man who had orchidopexy in childhood. It is still unclear why testicular cancer could recur in the scrotum after a radical orchidectomy.

## Introduction

Primary testicular cancer is the most common solid cancer in men aged 20-45 [1]. Comprising 1% of all male cancers and 5% of all urological tumors, testicular cancer is considered to be the most curable cancer [2]. More than 95% of testicular neoplasms are germ cell tumors (GCTs) [3]. GCTs are broadly divided into seminomatous and non-seminomatous due to differences in histology, natural history, and treatment [3], accounting for around 50% of cases each [2]. Non-seminomatous germ cell tumors (NSGCTs) are common in ages 20-35, while seminomatous is common in 35-45 years [1].

The incidence of testicular cancer is increasing in most European countries, but mortality has been on a steady decline since the introduction of platinum-based chemotherapy in 1975 [1].

Around 5%-10% of testicular cancer patients have a history of cryptorchidism. Cryptorchidism is associated with a three-fold increased risk of testicular cancer in men who underwent orchidopexy at age <13 years, but this risk is increased by six-fold in those who underwent orchidopexy at age >13 years [1]. Risk factors for testicular cancer are not well understood, but in addition to cryptorchidism, other factors such as age, abnormal testicular development, family history, ethnicity, genetic conditions (such as Down syndrome and Klinefelter syndrome) and being immunocompromised could all play a role [4]. Germ cell neoplasia in situ, or precursor lesions, can transform and become malignant cells, likely due to genetic and molecular changes, also contributing as a risk factor for GCTs [2].

Local recurrence rates for testicular cancer are 0.4% in inguinal orchidectomies and 2.9% for patients with scrotal violations [5]. Testicular cancer would normally present as a painless scrotal swelling.

We report the first case of ipsilateral local recurrence of NSGCT following radical orchidectomy in the literature.

## Case presentation

A man in his late twenties presented to the Accident and Emergency Department with a one-month history of ongoing right testicular pain and swelling. He was initially diagnosed and treated for epididymo-orchitis, but despite antibiotics, his symptoms persisted. He was then referred onward to the urology team for further management. He had a background history of bilateral orchidopexy for undescended testes in early childhood; he had no other medical problems and had investigations to rule out possible testicular cancer. 

On examination, he was found to have an enlarged right testicle and an atrophic left testicle. His tumor markers on admission were alpha fetoprotein (AFP) of 1710ng/mL, beta human chorionic gonadtropin (b-hCG) of 573µ/L and lactate dehydrogenase (LDH) of 257µ/L. He also had a testicular ultrasound scan, which showed a mass measuring 43x53x34mm. A regional MDT discussion was held, and a plan for right radical orchidectomy was recommended after sperm banking.

The following month, after his initial presentation, he had a right radical inguinal orchidectomy under general anesthesia. The testis was resected from the scrotal skin intact, with no violation of the testis whatsoever and no scrotal skin perforation. His histology report revealed a non-seminomatous germ cell tumor (95% yolk sac carcinoma (YST) and 5% embryonal carcinoma (EC), pT1). Following which, he was referred to our tertiary center for further management. He then had a staging CT scan, which was negative for any metastasis, and follow-up tumor markers were expectedly on the decline. His AFP was 18ng/mL and 6ng/mL after one and three months post-orchidectomy, respectively. 

**Figure 1:**
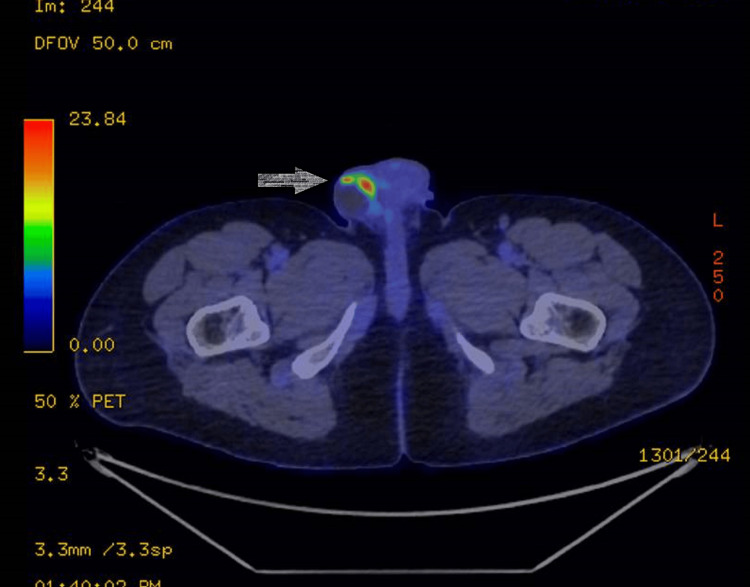
Whole-body FDG PET CT scan showing complex, partly-cystic, partly-solid mass at the right scrotum with intense FDG accumulation within solid components FDG: Fluorodeoxyglucose, PET: positron emission tomography, CT: computed tomography

**Figure 2:**
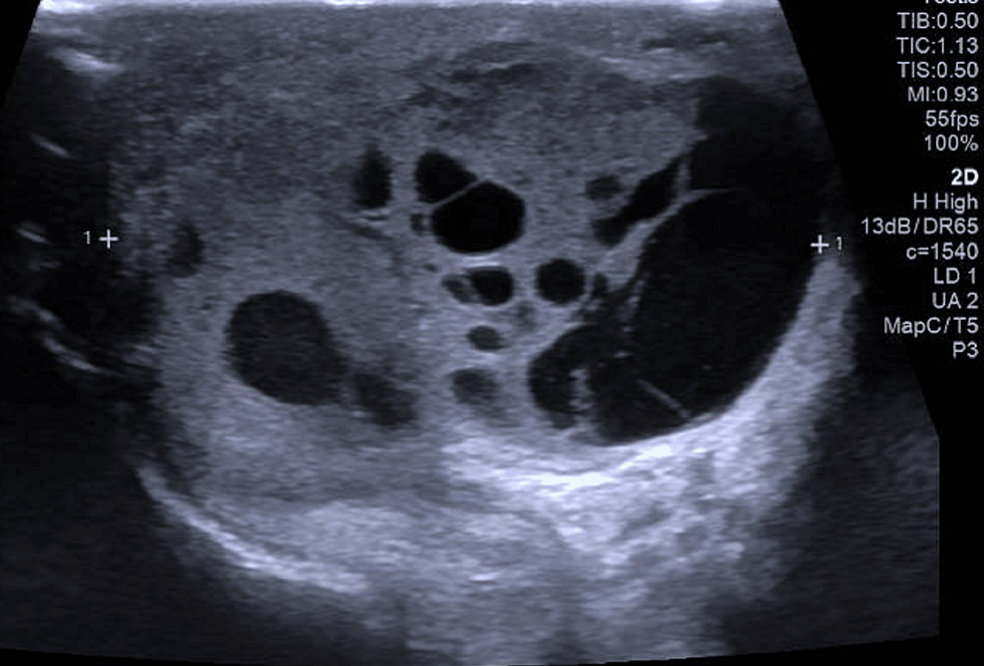
Ultrasound scan image showing a 44x27x37mm mixed solid/cystic mass with internal vascularity. within the right hemiscrotum.

Five months later, he presented with a new scrotal mass on the same side. The initial investigative ultrasound scan (Figure 2) showed a 44x27x37mm well-defined solid lobulated mass with similar testicular parenchyma whose exact nature was unknown. His tumor markers, particularly AFP, was notably elevated at 533ng/mL and LDH at 221U/L, along with new complains of some back pain. He went on to have a PET CT scan (Figure 1), which showed a complex hypermetabolic right scrotal mass, highly suspicious for residual or recurrent disease, with no FDG-avid extra scrotal disease identified. He was discussed at the MDT, following which he had a right hemiscrotectomy in less than four weeks from the new presentation with the mass. The histology showed a recurrent non-seminomatous germ cell tumor that was composed of yolk sac and embryonal carcinoma (yolk sac 60%, embryonal 40%). The tumor invaded the subcutaneous tissue of the scrotum and the overlying dermis. The tumor was 0.3mm from the nearest resection margin, with no evidence of neoplasia in the gubernaculum. He has recovered well post-operatively. His AFP was 12ng/mL and 7ng/mL after one and two months post-hemiscrotectomy, respectively. He received three cycles of BEP (bleomycin, etoposide, and cisplatin) adjuvant chemotherapy to reduce his risk of any further recurrences because his unusual recurrence puts him at high risk. He is still under surveillance and remains well.

## Discussion

We believe this case is the first reported occurrence of scrotal recurrence on the ipsilateral side post-orchidectomy for a NSGCT. As demonstrated in this case, past cryptorchidism can increase the risk of testicular cancer. The question arises: why was there a local recurrence post-orchidectomy?

A literature review was undertaken to consider cases of local scrotal recurrence post orchidectomy. A total of three papers were found to be relevant through systematic database searching on PubMed, Medline, and Embase. Papers were excluded if they were irrelevant to the search terms and if they were older than 15 years since publishing. From the amount and content of the included papers, it is evident that local scrotal recurrence of testicular cancer is rare after orchidectomy. There are only case reports within the last 10-15 years reporting such a phenomenon. Three case reports will be discussed within this section. 

The local recurrence of seminoma post-inguinal orchidectomy is rare, as reported by Hirose et al. in 2008 [5]. A 52-year-old man presented with a 9 x 5 cm right scrotal swelling with normal alpha fetoprotein but raised beta human chorionic gonadotropin markers. A right inguinal orchidectomy was performed, revealing a stage 1 testicular seminoma with no spermatic cord invasion but extensive invasion of the tunica albuginea. A follow-up CT scan three months post-surgery revealed a right-sided 5mm nodule that gradually became symptomatic, increasing to 30mm. The mass was resected 11 months post-orchidectomy, and histopathological examination revealed recurrent seminoma with negative surgical margins. A very old review reported that the local recurrence rates in 1,182 cases were 0.4% in inguinal orchidectomies [6], which is, therefore, incredibly rare. The prognosis of local recurrence for seminoma is unclear despite seminoma having a high survival rate [7]. The authors speculate malignant cells may have disseminated during the operation, resulting in local recurrence. The presentation is very similar to our case with the ipsilateral recurrence of NSGCT in the scrotum post-orchidectomy.

Ugwumba et al. [8] report a case of a 32-year-old male with a history of prior orchidectomy presenting with giant left hemi scrotal swelling. Previously, the patient had undergone a left orchidectomy three years prior for a mass; however, histology reports were unavailable. The presenting mass was giant, measuring 30 x 28 x 25cm and confined to the scrotum. This new mass was then inguinally resected and revealed to be a seminoma on histopathology reports. While an inguinal approach was taken for the orchidectomy, which is known to reduce local seeding and rupture of tumor cells [9], the authors speculate some ‘seed' areas of the tumor were missed in the first surgery, thus resulting in local recurrence. This theory of seeding of tumor cells from the original orchidectomy is agreed upon by Hirose et al. [5] and is more likely to be the cause of this case due to previous histology reports being unavailable, raising suspicion that surgical margins may not have been negative. Though these cases differ from our index case due to their presentation and sizes, tumor seeding appears to be a common theory for local recurrence post-odontectomy.

Similarly, Li et al. [10] presented a case of a local recurrence of testicular cancer in a non-violated scrotum. As mentioned, the inguinal approach for radical orchidectomy is preferred over scrotal manipulations to avoid the spread of disease, perhaps through lymphatic drainage disturbance and seeding of the tumor [11]. The presenting case was a 23-year-old male undergoing a right orchidectomy for a mixed non-seminomatous germ cell tumor (NSGCT), which then presented seven months later with a 1 cm left-sided scrotal mass. This mass was resected and analyzed to be a mature teratoma. However, this case is different from Ugwumba et al. and Hirose et al. because the patient, in addition to local recurrence on the adjacent left hemiscrotum, experienced the uncommon presentation of growing teratoma syndrome. The authors speculate similarly to Ugwumba et al. and Hirose et al., suggesting there could have been likely microscopic residual tumor cells in the scrotum at original surgery, despite pathology documents reporting negative surgical margins. While the case reported by Li et al. is very similar to ours, the main differences are that ours is presented on the ipsilateral side for recurrence, and there is nothing to suggest a growing teratoma syndrome.

The main working theory appears to be that while local recurrence post-orchiectomy appears to be incredibly rare, all three case reports speculate residual cells from the original orchidectomy to be the reason for local recurrence. Additionally, Li et al. recommend that patients be closely surveyed post-treatment to avoid missing any abnormalities, especially since the prognosis is unclear in this cohort of patients.

## Conclusions

It has been clearly documented that undescended testis, corrected or not, predisposes to testicular cancer. Childhood orchidopexy for undescended testis appears to be a risk for testicular cancer, as demonstrated by our case, which can result in a local recurrence of the same NSGCT after inguinal orchidectomy. While the literature shows that local recurrence after inguinal orchidectomy is incredibly rare, there is still a risk of some cells remaining despite maintaining negative surgical margins, which can predispose to recurrence. In the case presented on the ipsilateral scrotum for local recurrence, other reports have demonstrated that this recurrence can be on the contralateral side as well. However, the reason for this pattern of dissemination remains unclear. We advise that patients should be closely monitored post-odontectomy to monitor the chance of local recurrence on either scrotum, despite its rarity, and treated following a MDT discussion.
